# Uraemic extracellular vesicles augment osteogenic transdifferentiation of vascular smooth muscle cells via enhanced AKT signalling and PiT‐1 expression

**DOI:** 10.1111/jcmm.16572

**Published:** 2021-05-07

**Authors:** Christian Freise, Uwe Querfeld, Antje Ludwig, Bernd Hamm, Jörg Schnorr, Matthias Taupitz

**Affiliations:** ^1^ Department of Radiology Charité – Universitätsmedizin Berlin, corporate member of Freie Universität Berlin, Humboldt‐Universität zu Berlin, and Berlin Institute of Health Berlin Germany; ^2^ Department of Pediatric Gastroenterology, Nephrology and Metabolic Diseases Charité – Universitätsmedizin Berlin, corporate member of Freie Universität Berlin Humboldt‐Universität zu Berlin, and Berlin Institute of Health Berlin Germany; ^3^ Department of Cardiology and Angiology Charité – Universitätsmedizin Berlin, corporate member of Freie Universität Berlin, Humboldt‐Universität zu Berlin, and Berlin Institute of Health Berlin Germany

**Keywords:** chronic kidney disease, exosomes, extracellular vesicles, uraemia, vascular calcification, vascular smooth muscle cells

## Abstract

Extracellular vesicles (EV) function as messengers between endothelial cells (EC) and vascular smooth muscle cells (VSMC). Since chronic kidney disease (CKD) increases the risk for vascular calcifications, we investigated whether EV derived from uraemic milieu‐stimulated EC and derived from uraemic rats impact the osteogenic transdifferentiation/calcification of VSMC. For that purpose, human EC were treated with urea and indoxyl sulphate or left untreated. Experimental uraemia in rats was induced by adenine feeding. ‘Uraemic’ and control EV (EV^UR^; EV^CTRL^) were isolated from supernatants and plasma by using an exosome isolation reagent. Rat VSMC were treated with a pro‐calcifying medium (CM) with or without EV supplementation. Gene expressions, miRNA contents and protein expressions were determined by qPCR and Western blots, respectively. Calcifications were determined by colorimetric assays. Delivery of miRNA inhibitors/mimics to EV and siRNA to VSMC was achieved via transfection. EV^CTRL^ and EV^UR^ differed in size and miRNA contents. Contrary to EV^CTRL^, EC‐ and plasma‐derived EV^UR^ significantly increased the pro‐calcifying effects of CM, including altered gene expressions of osterix, runx2, osteocalcin and SM22α. Further, EV^UR^ enhanced the protein expression of the phosphate transporter PiT‐1 in VSMC and induced a phosphorylation of AKT and ERK. Knock down of PiT‐1 and individual inhibition of AKT and ERK signalling in VSMC blocked the pro‐calcifying effects of EV^UR^. Similar effects were achieved by inhibition of miR‐221/‐222 and mimicking of miR‐143/‐145 in EV^UR^. In conclusion, EV^UR^ might represent an additional puzzle piece of the complex pathophysiology of vascular calcifications in CKD.

## 1 INTRODUCTION

A high‐risk group for the development of extensive and accelerated vascular calcifications are patients with chronic kidney disease (CKD), including children.^1^, ^2^, ^3^ Their impaired renal function and subsequently increased plasma levels of uraemic toxins and elevated phosphate and calcium levels contribute to vascular calcification via multiple mechanisms.^4^

Even today, the pathophysiology of vascular calcifications such as arteriosclerosis is still not fully understood and there is no established therapy for the inhibition of onset and progression of vascular calcifications.

Extracellular vesicles (EV) and their (patho) physiological functions came into the focus of cardiovascular research during the last years.^5^, ^6^ Cells release EV under both physiological and pathophysiological conditions. Subtypes of EV differ in their cellular origin, size, membrane structure and their cargo load. Two important EV subtypes are microvesicles (size ~100‐1000 nm) and exosomes (~20‐150 nm).^7^

Exosomes were shown to impact the calcification of vascular smooth muscle cells (VSMCs),^2^, ^8^ a key event during the development of arteriosclerosis.^9^ Pro‐calcifying conditions can induce the generation of pro‐calcifying exosomes in VSMC^10^ and calcified VSMCs in turn secrete exosomes which promote calcification of non‐calcified VSMCs,^11^ thus promoting progression of vascular calcification.

Besides this autocrine stimulation, also a paracrine exchange of exosomes, for example, between endothelial cells (EC) and VSMC is known, which contributes to the communication between cells.^12^, ^13^ For instance, EC‐derived exosomes could impact the phenotype of VSMC.^12^ Thus, exosomes function as messengers in the crosstalk of endothelial and smooth muscle cells in the arterial wall.^14^ Importantly, the release of EV by EC is also a function of the surrounding milieu, for example, the presence of cardiovascular risk factors like tumour necrosis factor‐α (TNF‐α) or angiotensin II.^15^, ^16^

EC are in constant contact with the blood stream and interact with its components. The uraemic milieu in CKD promotes constant injury to the endothelium by inflammatory cytokines, uraemic toxins, urea and other deleterious signals.^17^ We therefore have been suggested that uraemic toxin‐stimulated EC produce EV, which augment the calcium‐ and phosphate‐induced osteogenic transdifferentiation/calcification of adjacent VSMC.

## 2 MATERIALS AND METHODS

### 2.1 Cell culture

Human endothelial cells (EA. Hy926, ATCC^®^ CRL‐2922™) and rat aortic VSMC (A7r5, ATCC® CRL‐1444™) were routinely cultured in standard culture medium which consisted of DMEM (Gibco/Thermo Fisher Scientific, Hennigsdorf, Germany) with 862 mg/L L‐alanyl‐l‐glutamine, 1.0 g/L glucose, 50 μg/mL streptomycin, 50 units/ml penicillin and 10% heat‐inactivated foetal bovine serum (FBS, Gibco). To avoid FBS‐specific differences in the calcification experiments, FBS was used from the same lot throughout the whole study. Cells were cultured in a humidified atmosphere at 37°C and 5% CO_2_. Cells were maintained at 70%‐80% confluence by passaging as needed.

### 2.2 Treatment of endothelial cells with uraemic toxins

To mimic uraemic conditions in vitro, EC were treated for 7 days in 75 cm^2^ flasks (NUNC) with standard culture medium supplemented with 20 mmol/L urea (Merck) which roughly corresponds to concentrations seen during chronic renal failure^18^ and 375 µmol/L (50 µg/mL) indoxyl sulphate (IS; Sigma‐Aldrich) which corresponds to mean plasma concentrations in uraemic patients.^19^ The medium was replaced every 2nd day. Untreated cells served as control.

### 2.3 Rat model of chronic kidney disease

Chronic kidney disease in rats was induced by adenine feeding. Details are described in the Appendix S1. Serum chemistry characteristics of the experimental groups are given in Table S1.

### 2.4 Isolation of extracellular vesicles from cell culture supernatants and plasma from rats

EV derived from EC treated with uraemic toxins and from uraemic rats are referred to hereinafter as uraemic EV (EV^UR^), while EV from control treated EC and control rats are termed control EV (EV^CRTL^). A flow chart of the isolation procedure is shown in Figure S1. The detailed isolation procedure is described in the Appendix S1.

### 2.5 Characterization of extracellular vesicles

EVs were further characterized by dynamic light scattering, FACS analysis, Western blot and miRNA contents by RT‐PCR as described in the Appendix S1.

### 2.6 Treatment of VSMC with calcium, phosphate ± EV^UR^/EV^CTRL^

Vascular smooth muscle cells were grown in 24‐well plates or 6‐well plates (NUNC) for 24 h (day 0). After reaching ~90% confluence, the culture medium was replaced by a calcification medium (CM) which consisted of standard culture medium supplemented with calcium chloride (2.7 mmol/L vs. 1.8 mmol/L) and sodium dihydrogen phosphate (2.8 mmol/L vs. 1.0 mmol/L). CM was changed every second day. To study specific effects of EV from EC culture supernatants, CM and the control medium were supplemented with 150 µg/mL EV^UR^ or EV^CTRL^, respectively. Effects of rat plasma‐derived EV were investigated in an analogous manner. In order to save material, a concentration of 150 µg/mL represented a reasonable compromise regarding the ratio of EV‐mediated effects and the necessary amounts of EV.

### 2.7 Analysis of treatment‐dependent calcifications in VSMC cultures

The degree of mineralization of VSMC cultures was determined after 5 or 7 days of treatment by Alizarin‐red staining as described.^20^ Enzymatic activities of alkaline phosphatase (ALP) were determined in supernatants of the VSMC cultures,^20^ and calcium contents of the cultures were analysed by the o‐cresolphthalein complexone (OCPC) method.^20^ Calcium contents and ALP levels were normalized to the protein contents as determined by the BCA method.

### 2.8 Analysis of gene and protein expressions in VSMC

The respective passages describing the measurements of gene and protein expressions in VSMC can be found in the Appendix S1.

### 2.9 Inhibition of AKT and ERK phosphorylation in VSMC

We applied LY3023414 (Ly30; Selleckchem, Munich, Germany), a PI3K/AKT/mTOR inhibitor, to interfere with EV^UR^‐induced activation of AKT in VSMC. The activation of ERK1/2 in VSMC was blocked by applying the inhibitor UO126 (Cell signalling). In Western blot experiments, the cells were pre‐incubated with 50 nmol/L Ly30 or 10 µmol/L UO126 for 5 minutes prior to the respective treatment. Both inhibitors were dissolved in DMSO and the resulting maximum DMSO concentration in the experiments was 0.05%. To avoid long‐term toxicity of the inhibitors, the VSMC were treated prior to every medium change (every 2nd day) for 5 minutes with Ly30 or UO126.

### 2.10 Transfection of EV and VSMC

Isolated EV^UR^/EV^CTRL^ were transfected with miR inhibitors, miR mimics or respective controls using the Exo‐Fect™ siRNA/miRNA Transfection Kit (System Biosciences, Palo Alto, CA, USA) according to the manufacturer's instructions. All used miR inhibitors and miR mimics are given in Table S2. 24 hours after transfection, the EV were used in the respective experiments.

VSMC were transfected with siRNA targeted against PiT‐1 (Silencer™ Slc20a1 siRNA, #AM16708, Invitrogen™) or a respective negative control (Silencer™ Negative Control No. 1 siRNA, #AM4611, Invitrogen™) using the HiPerFect Transfection Reagent (Qiagen) according to the manufacturer's instructions.

### 2.11 Statistical analyses

Data sets were analysed by one‐way or two‐way ANOVA followed by the Tukey post hoc test using GraphPad PRISM, version 6.01 (GraphPad Software), and differences with *P*‐values <.05 (*) were considered statistically significant.

## 3 RESULTS

### 3.1 Characterization of EC‐derived EV^UR^ and EV^CTRL^

Using the EC supernatants, the isolation procedure yielded two EV fractions for each treatment group (control vs. uraemic toxins), a putative exosome‐containing fraction and a putative microvesicle‐containing fraction (Figure S1). In this study, we focused on the putative exosome‐containing fraction only. Dynamic light scattering analysis revealed different hydrodynamic diameters of 53.2 ± 13.9 nm and 72.5 ± 8.0 nm (*P* <.05) for EV^CTRL^ and EV^UR^, respectively (Figure S2A). A concomitant FACS analysis of both groups from the ‘exosome’‐fraction revealed an intense signal for the two exosome markers CD9 and CD81. In comparison, the respective signals in the putative ‘microvesicle’ fractions were distinctly reduced (Figure S2B). Further, both ‘exosome’‐fractions but not the corresponding ‘microvesicle’‐fractions contained the exosome marker Alix, as shown by Western blot analysis (Figure S2C).

### 3.2 EV^UR^ augment calcium‐ and phosphate‐induced morphological and transcriptional changes in VSMC

**FIGURE 1:**
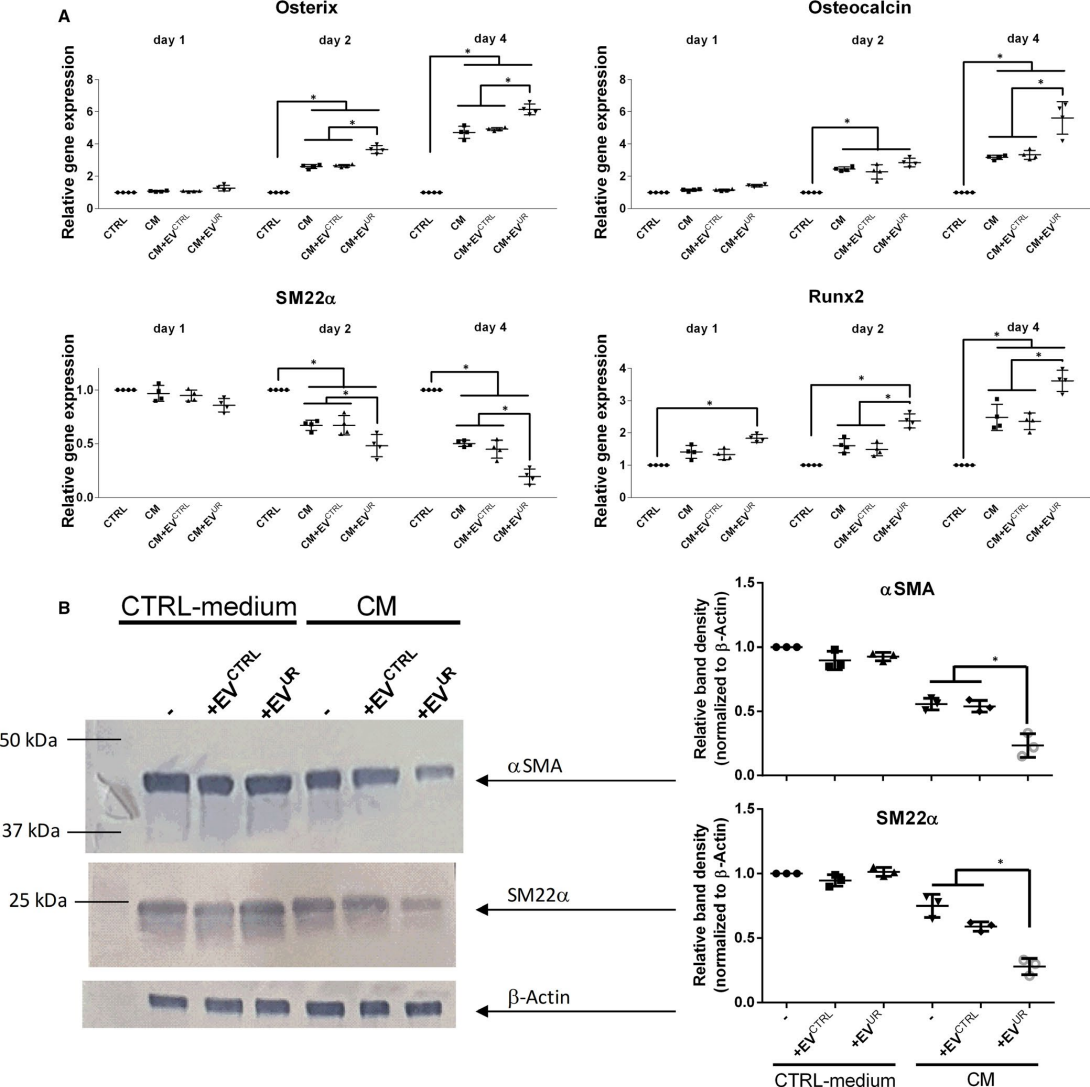
EV^UR^ augment calcium‐ and phosphorous‐induced transcriptional changes in VSMC. A, Gene expressions in VSMC were determined by qPCR after treatment of the cells as indicated. Shown are means ± SD (n = 4; *P < .05). B, Rat VSMC were treated as indicated for 5 days. Effects on protein expressions were determined by Western blot. Displayed are representative blots together with means ± SD from quantification of respective band intensities (n = 3)

As expected from previous studies, treatment with CM alone induced a significant up‐regulation of osterix, osteocalcin and runx2 after 2 and 4 days compared to control, while the gene expression of SM22α was significantly reduced (Figure 1A). Supplementation of CM with EV^UR^ significantly enhanced the gene expressions of osterix and runx2 after 2 and 4 days compared to treatment with CM alone and gene expressions of osteocalcin and SM22α were significantly enhanced/reduced after 4 days (Figure 1A). Of note, the gene expression of runx2 was significantly enhanced by EV^UR^ already after 24 hours compared to CM alone.

In line with the gene expression data, CM induced slight morphological changes of VSMC within 72 hours compared to control (Figure S3A). The presence of EV^UR^ distinctly enhanced and accelerated the effects of CM on morphology. After 48 hours, distinct signs of mineral depositions were visible, which were massively enhanced after 72 hours (Figure S3A). In contrast, EV^CTRL^ provoked no obviously different morphological changes compared to treatment with CM alone. When applied in control medium, neither EV^CTRL^ nor EV^UR^ induced any morphological changes compared to control (Figure S3B), implicating that the effects of EV^UR^ require the presence of elevated concentrations of phosphate and calcium.

Like the effects on cell morphology, neither EV^CTRL^ nor EV^UR^ significantly affected the gene expressions of the above‐mentioned marker genes when applied in standard culture medium (Figure S3C).

The effects of EV^UR^ on SM22α expression were confirmed also on protein level by Western blot analysis further complemented by an analysis of α‐SMA. Treatment with CM for 5 days reduced the protein expression of SM22α by ~20% compared to control. This effect was strongly enhanced by EV^UR^ (reduction of ~70%), while EV^CTRL^ only slightly impacted the effects of CM on SM22α expression (Figure 1 B). For α‐SMA, we observed comparable effects. CM induced a reduction of α‐SMA expression by ~50%, whereas EV^UR^ enhanced these effects by up to ~80% (Figure 1 C).

### 3.3 EV^UR^ augment calcium‐ and phosphate‐induced mineralization of VSMC cultures

**FIGURE 2:**
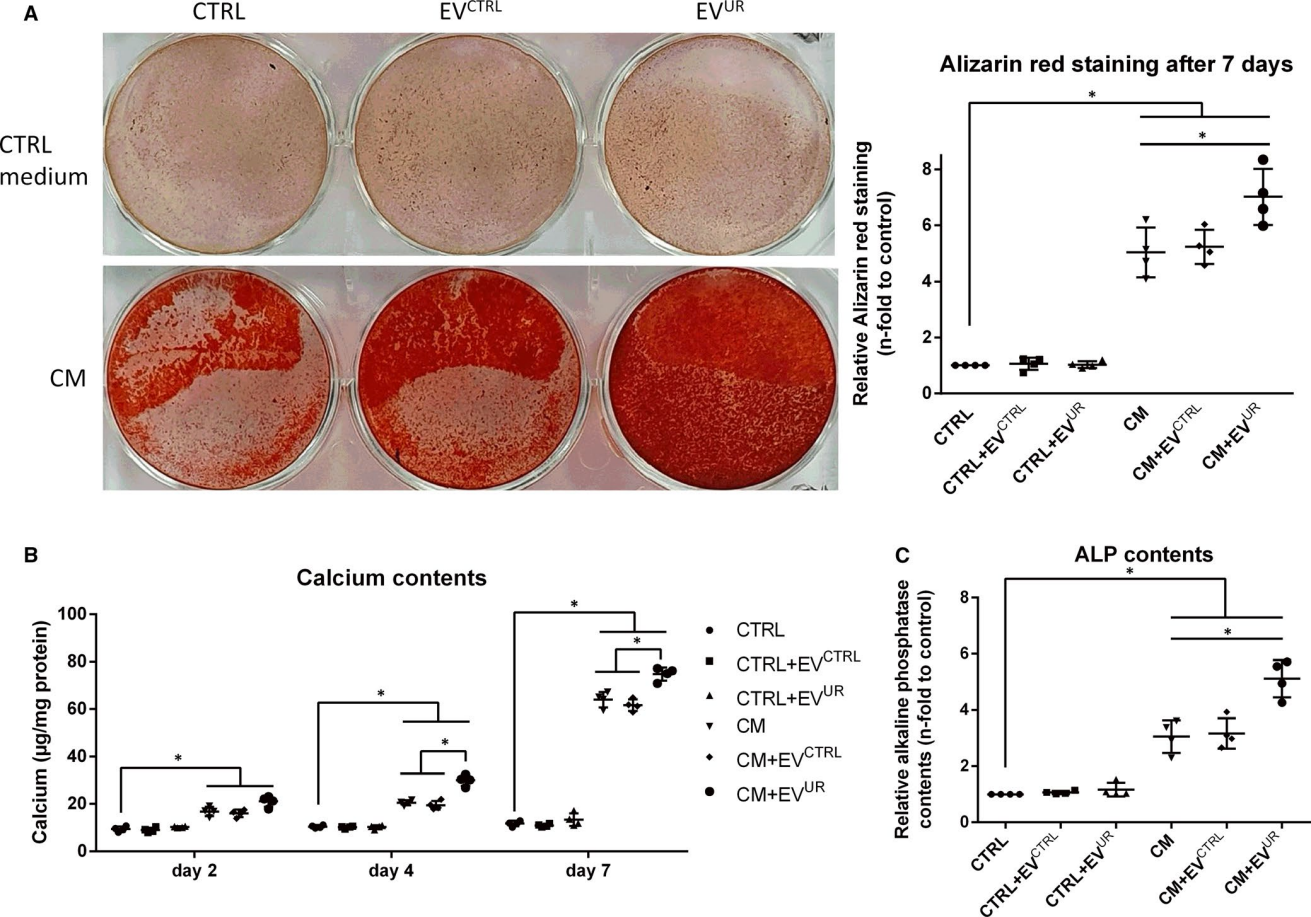
EV^UR^ augment calcium‐ and phosphate‐induced calcification on VSMC. A, VSMC were treated as indicated for 7 days. Shown are representative images of cells stained with Alizarin‐red and data on quantification of bound Alizarin‐red dye. Shown are means ± SD (n = 4). In parallel set ups. B, calcium contents of the cultures were determined by the o‐cresolphtalein method and C, alkaline phosphatase contents were determined in the supernatants using the Quanti‐Blue^TM^ reagent. Shown are means ± SD. (n = 4, *P <.05)

To confirm that the observed morphological and transcriptional changes of VSMC lead to mineralized VSMC cultures, Alizarin‐red staining and calcium measurements were performed. When cultured in CM, the presence of EV^UR^ significantly increased mineral deposition in VSMC compared to CM alone, shown by Alizarin‐red staining and calcium measurements (Figure 2A,B). EV^CTRL^ provoked no distinct differences compared to CM alone (Figure 2A,B). Cells treated with control medium exhibited no obvious calcifications regardless of the presence of EV^CTRL^ or EV^UR^ (Figure 2A,B). Complementing the hitherto data, VSMC treated with CM+EV^UR^ showed higher ALP activities in supernatants than cells treated with CM only (Figure 2C).

In parallel, we investigated whether the stimulating effects of EV^UR^ on VSMC calcification are because of the induction of apoptosis. However, neither EV^CTRL^ nor EV^UR^ induced stronger caspase‐3/‐7 activities compared to treatment with CM alone (Figure S4).

### 3.4 EV^UR^ promote activation of AKT and ERK signalling and induce an enhanced expression of PiT‐1 in VSMC

**FIGURE 3:**
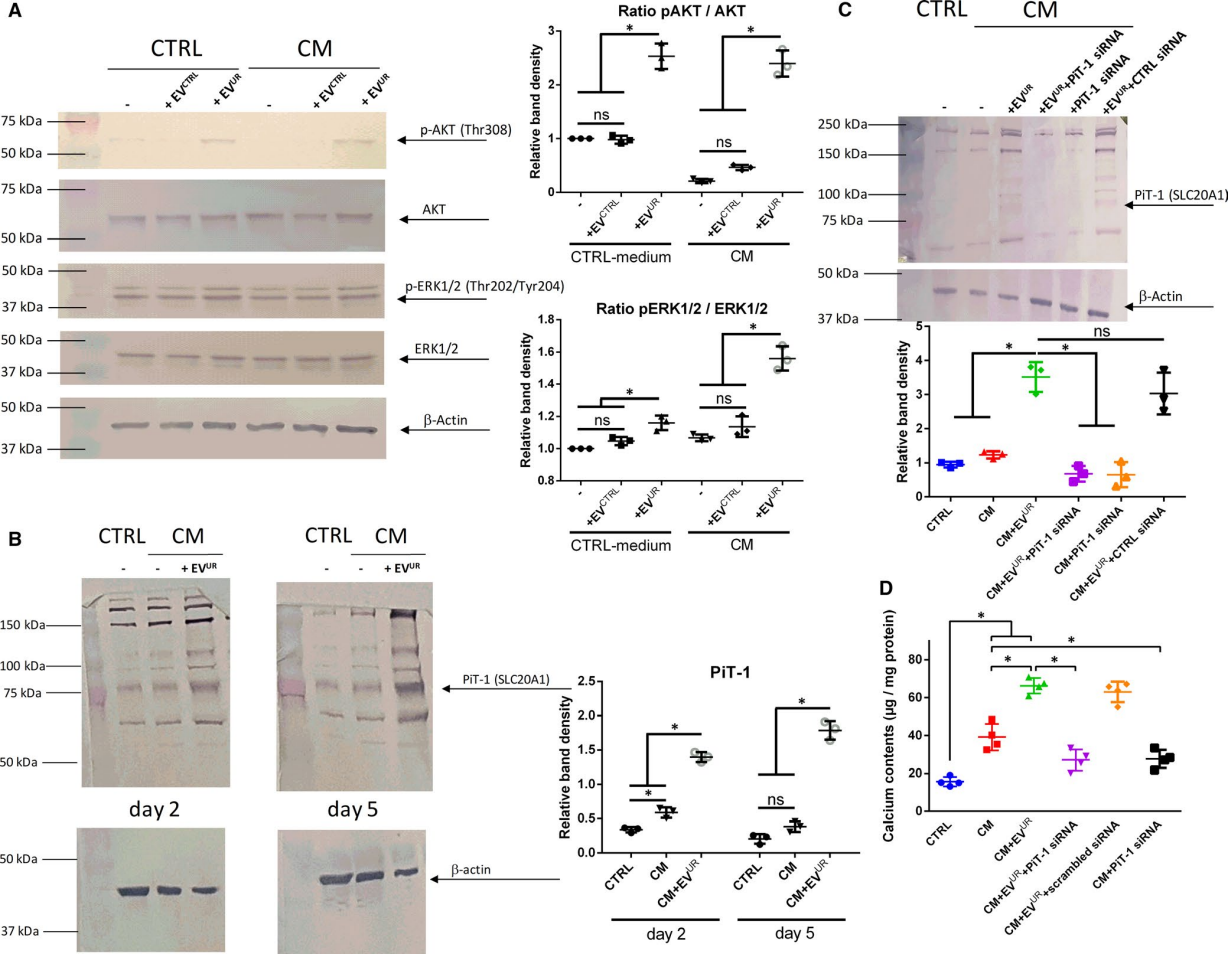
EV^UR^ promote activation of AKT and ERK signalling and induce an enhanced expression of PiT‐1 in VSMC. VSMC were cultured and treated in 6‐well microtitre plates as indicated. Shown are treatment‐dependent effects on (A) the phosphorylation of ERK1/2 and of AKT after 15 min. In addition, treatment‐dependent effects on (B) the protein expression of PiT‐1 after 2 and 5 days of treatment are shown. Displayed are representative results from one out of three independent experiments along with means ± SD from quantification of respective band intensities. *P <.05. C, Protein expression of PiT‐1 in VSMC was knocked down by siRNA. Shown is one representative Western blot out of three along with means ±SD from quantification of respective band densities (n = 3, *P <.05). D, In parallel, calcium contents of the cultures were determined by the o‐cresolphtalein method. Shown are means ± SD. (n = 4, *P <.05)

To gain first insights into underlying mechanisms of EV^UR^ in VSMC, we analysed AKT and ERK signalling which both are known to impact calcification of VSMC.^21^ When treated in standard culture medium, EV^UR^ induced a strong phosphorylation of AKT after 15 minutes, while EV^CTRL^ showed no effects (Figure 3A). A similar result was observed when using CM. Only the presence of EV^UR^ strongly phosphorylated AKT (Figure 3A). For the phosphorylation of ERK1/2, effects of EV^UR^ were also evident and enhanced compared to the respective controls in standard culture medium as well as in CM, but the effects were less pronounced compared to the EV^UR^‐mediated phosphorylation of AKT (Figure 3A).

Since treatment with EV^CTRL^ did not result in significantly different effects in the cells compared to treatment with CM alone, we hereinafter focused on the effects of EV^UR^ only.

AKT and ERK signalling are closely linked to the induction of the sodium‐dependent phosphate co‐transporter PiT‐1 in calcifying VSMC and PiT‐1 in turn is known to contribute to the calcification of VSMC cultures.^21^, ^22^ Therefore, we subsequently studied effects of EV^UR^ on the expression of PiT‐1. Treatment of VSMC with CM induced a higher PiT‐1 expression compared to control after 2 and 5 days of treatment (Figure 3B). The supplementation of EV^UR^ significantly enhanced the protein expression of PiT‐1 compared to treatment with CM alone after 2 and 5 days (Figure 3B).

We consequently applied PiT‐1 siRNA to selectively knock down the PiT‐1 protein expression in VSMC. Indeed, PiT‐1 siRNA strongly reduced the protein expression of PiT‐1 in VSMC treated with CM+EV^UR^, while an unspecific control siRNA did not show any effects (Figure 3C). Further, a knock down of PiT‐1 significantly reduced the pro‐calcifying effects of EV^UR^ indicated by strongly reduced calcium depositions in VSMC treated with CM and CM+EV^UR^ (Figure 3D). Again, a control siRNA did not reduce the calcium contents of the VSMC cultures (Figure 3D).

### 3.5 Inhibition of AKT and ERK signalling alleviates the facilitating effects of EV^UR^ on calcium‐ and phosphate‐induced mineralization of VSMC

**FIGURE 4:**
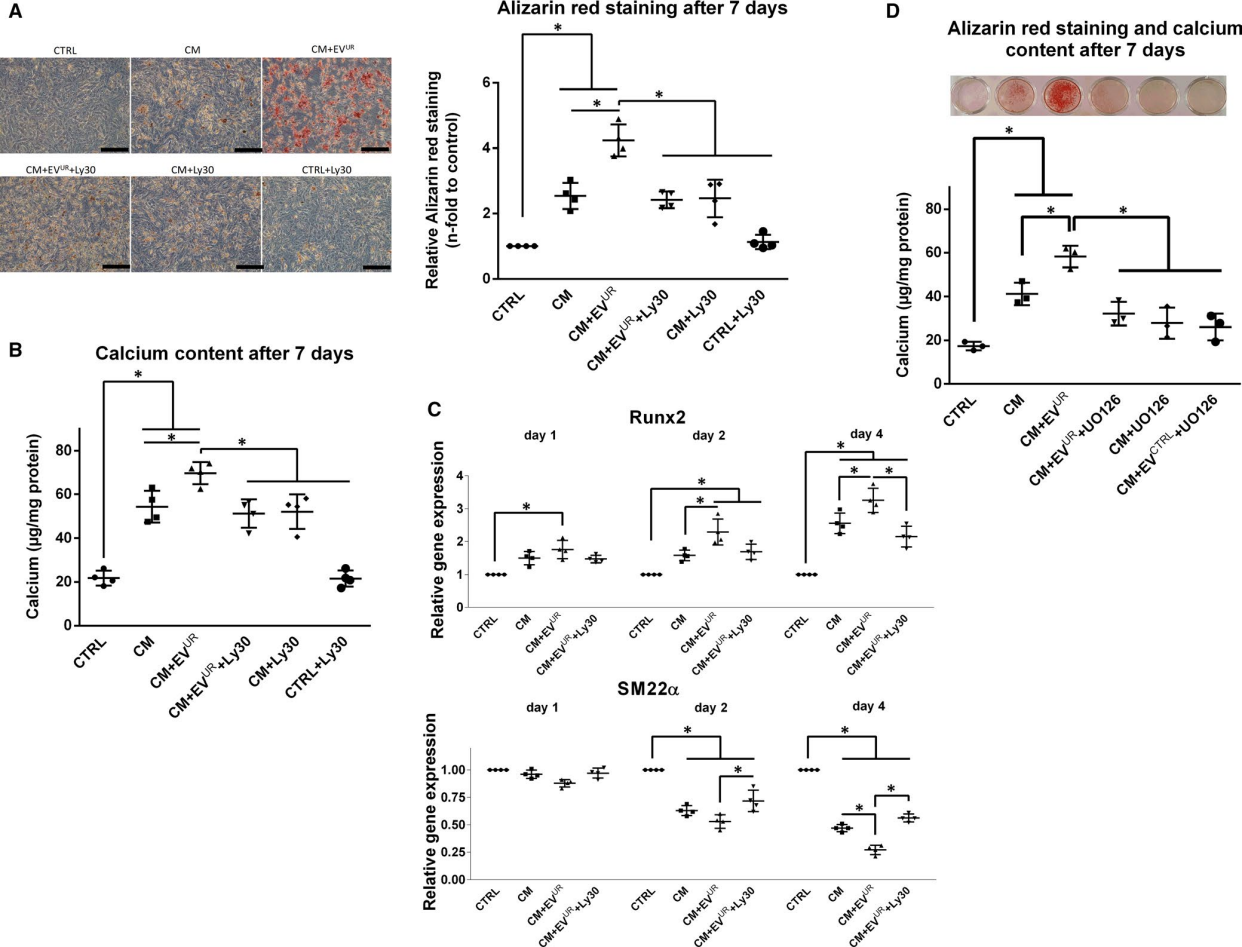
Inhibition of AKT‐ and ERK signalling attenuate EV^UR^‐mediated effects on VSMC calcification. A, VSMC were cultured like in the calcification experiments with or without periodic treatment with the PI3K/AKT/mTOR inhibitor Ly3023414 (Ly30). After 7 days, the cells were stained with Alizarin‐red. Shown are representative images of stained cells together with means ± SD from quantification of the red dye (n = 4, bars = 100 µm). B, In a parallel set up, calcium contents of the cultures were determined by the o‐cresolphtalein method (n = 4). C, Effects of Ly30 on the osteogenic transdifferentiation of VSMC were determined by qPCR after treatment of the cells as indicated. Shown are means ± SD (n = 4; *P <.05). D, VSMC were cultured like in the calcification experiments with or without periodic treatment with the MEK inhibitor UO126. After 7 days, the cells were stained with Alizarin‐red. Shown are representative images of stained cells (n = 3). In parallel, calcium contents of the cultures were determined by the o‐cresolphtalein method. Shown are means ± SD (n = 3; *P <.05)

Since the activation of AKT signalling appears to be a target of EV^UR^ in VSMC, we applied the AKT inhibitor Ly30 in our in vitro calcification model. Alizarin‐Red staining experiments revealed protective effects of AKT inhibition against the augmented calcification inducing effects of EV^UR^ compared to CM alone (Figure 4A). Of note, the AKT inhibitor did not prevent VSMC from CM‐induced calcifications. These data were confirmed by parallel measurements of the calcium contents in the VSMC cultures (Figure 4B). On transcriptional level, the above shown strong induction of runx2 expression by CM+EV^UR^ was distinctly alleviated after 1 and 2 days and significantly reduced after 4 days of treatment (Figure 4C). Further, AKT inhibition significantly reduced the EV^UR^‐induced decline of SM22α expression after 2 and 4 days of treatment (Figure 4C). Comparable results were observed after inhibition of ERK signalling in VSMC. The promoting effects of EV^UR^ on VSMC calcifications were neutralized by the synthetic inhibitor UO126 (Figure 4D).

### 3.6 EV^UR^ and EV^CTRL^ differ in their miRNA loading and targeted manipulation of miRNA activities impacts the pro‐calcifying effects of EV^UR^

**FIGURE 5:**
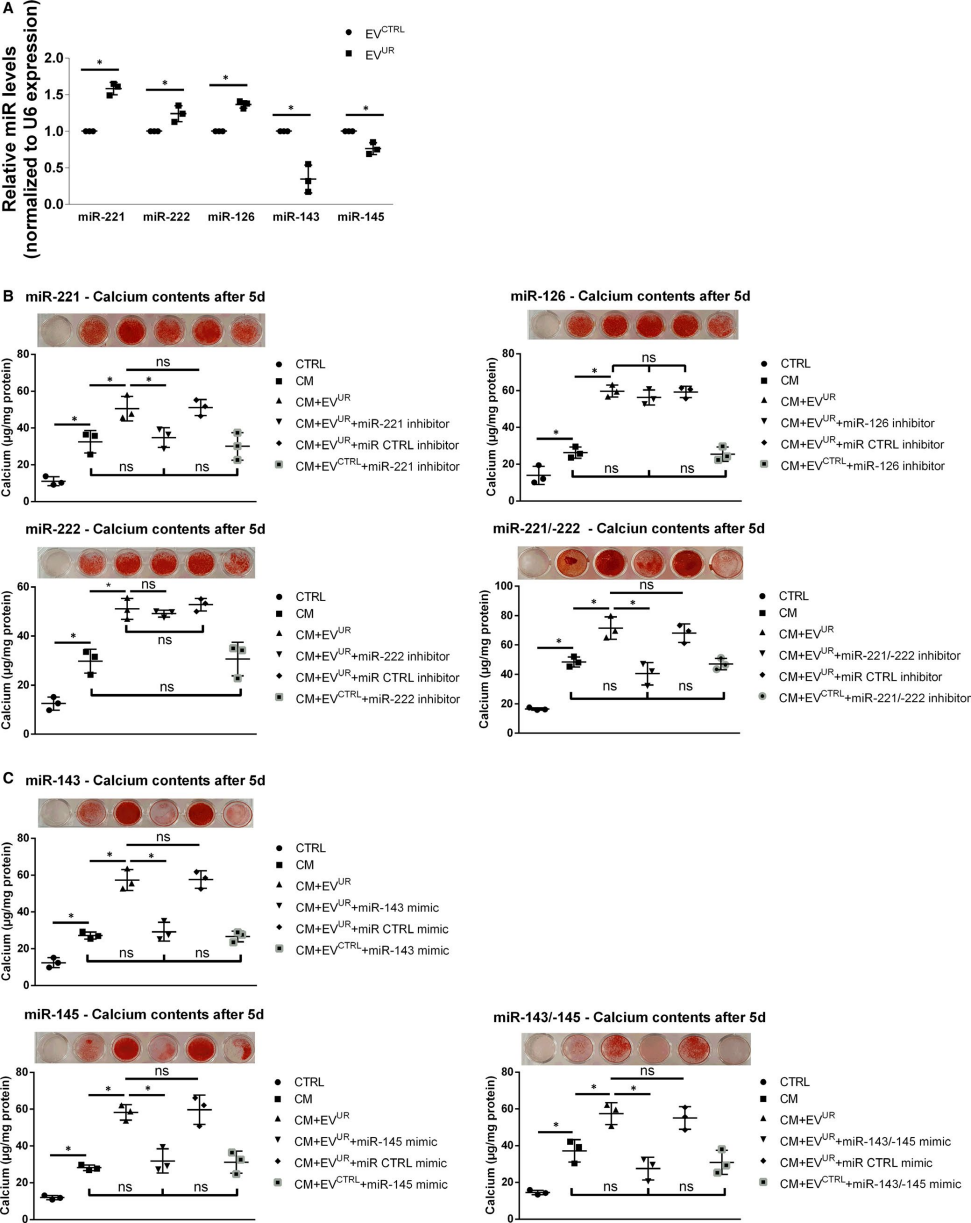
miRNA contents affect the pro‐calcifying effects of EV^UR^ on VSMC. A, The contents of five preselected miRNA (miR) in the EV were analysed by qRT‐PCR. Shown are means ± SD from three independent measurements. *P <.05. B, C, EV^UR^ were transfected with miR inhibitors, miR mimics or respective negative controls. After 24 h, the modified EV^UR^ were applied in VSMC calcification experiments. Mineralization of the cultures was determined by the o‐cresolphtalein method (means ± SD; n = 3, *P <.05) and by Alizarin‐red staining (representative images; n = 3)

EV are known to function as ‘shuttles’, for example, for miRNAs (miR). Therefore, we additionally analysed the contents of five preselected miR in EV^CTRL^ and EV^UR^ from the ‘exosome’‐fractions. All selected miR are known to be expressed in EC and to impact vascular calcification. Three miRNAs were significantly enhanced in EV^UR^ compared to EV^CTRL^. The differences for miR‐221 were the strongest (1.55 ± 0.08‐fold enhanced, *P* < .05) followed by miR‐126 (1.37 ± 0.05, *P* < .05) and miR‐222 (1.24 ± 0.11, *P* < .05) (Figure 5A). In contrast, the levels of mir‐143 and miR‐145 were significantly reduced in EV^UR^ compared to EV^CTRL^ (0.35 ± 0.19, *P* < .05 and 0.76 ± 0.08, *P* < .05, respectively) (Figure 5A). To gain insights into their potential functional relevance for the pro‐calcifying effects of EV^UR^, we applied specific miR inhibitors or miR mimics. Their successful transfection into exosomes was validated by qRT‐PCR measurements (Figure S5). Indeed, transfection of EV^UR^ with a miR‐221 inhibitor provoked a distinct reduction of VSMC calcifications while the inhibition of miR‐126 or miR‐222 provoked no attenuating effects (Figure 5B). A simultaneous inhibition of miR‐221 and miR‐222 tended to cause a stronger reduction of the pro‐calcifying effects of EV^UR^ than by inhibition of miR‐221 alone (Figure 5B).

Since levels of mir‐143 and miR‐145 were reduced in EV^UR^, we applied miR mimics to selectively enhance their activity in EV^UR^. An individual transfection with miR‐143 or miR‐145 mimics provoked a distinct reduction of VSMC calcifications (Figure 5C). Like miR‐221/‐222, a simultaneous approach tended to be even more effective in reducing the pro‐calcifying effects of EV^UR^ (Figure 5C).

For all miR, transfection of EV^UR^ with the negative controls for miR inhibitors or miR mimics provoked no differences in VSMC calcification compared to treatment with non‐transfected EV^UR^. Further, treatment with transfected EV^CTRL^ provoked no differences to treatment with CM alone (Figure 5B,C).

### 3.7 Plasma‐derived exosome‐like EV from uraemic rats promote VSMC transdifferentiation ex vivo

**FIGURE 6:**
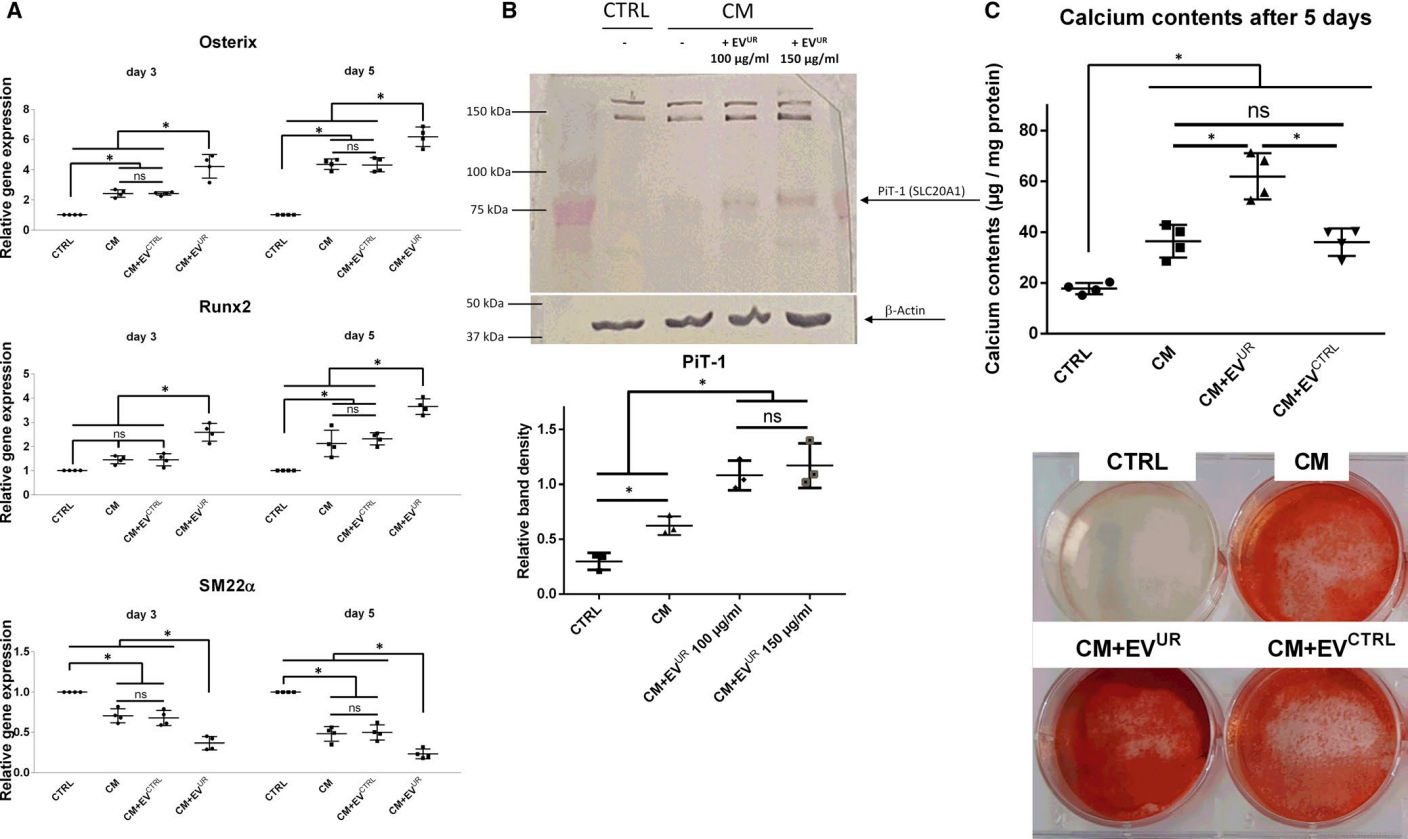
Plasma‐derived exosome‐like EV from uraemic rats promote VSMC transdifferentiation ex vivo. VSMC were cultured as indicated. A, Gene expressions in VSMC were determined by qPCR. Shown are means ± SD (n = 4). B, Treatment‐dependent effects of EV^UR^ on the protein expression of PiT‐1 after 3 days are shown. Displayed are representative results from one out of three independent experiments along with means ± SD from quantification of respective band intensities. Band densities were normalized to β‐actin. C, Parallel to the qPCR measurements, mineralization of the cultures was determined by the o‐cresolphtalein method (means ± SD; n = 4) and by Alizarin‐red staining (representative images; n = 4). *P <.05, ^ns^not significant

To finally investigate whether our experimental approach could in principle be translated to subsequent in vivo studies, we applied plasma‐derived EV from uraemic rats and healthy controls in our calcification model in vitro. A comparison of both EV fractions revealed hydrodynamic diameters of 43.8 ± 2.3 nm for EV^UR^ and 32.7 ± 1.9 nm for EV^CTRL^. Like the in vitro‐derived EV^UR^ from EC, the in vivo‐derived EV^UR^ but not EV^CTRL^ enhanced the CM‐induced transdifferentiation of VSMC. The supplementation with EV^UR^ significantly enhanced gene expressions of runx2 and osterix compared to treatment with CM alone, while the expression of SM22a was strongly decreased (Figure 6A). Furthermore, cells treated with CM+EV^UR^ exhibited significantly enhanced PiT‐1 protein expression levels after 3 days than cells treated with CM only (Figure 6B). This was accompanied by a significantly enhanced mineralization of VSMC cultures indicated by enhanced calcium levels and Alizarin‐red staining after 5 days compared to treatment with CM alone. EV^CTRL^ showed no such stimulatory effects (Figure 6C,D).

### 3.8 Inhibition of AKT blocks pro‐calcifying effects of EV^UR^ from uraemic rats

**FIGURE 7:**
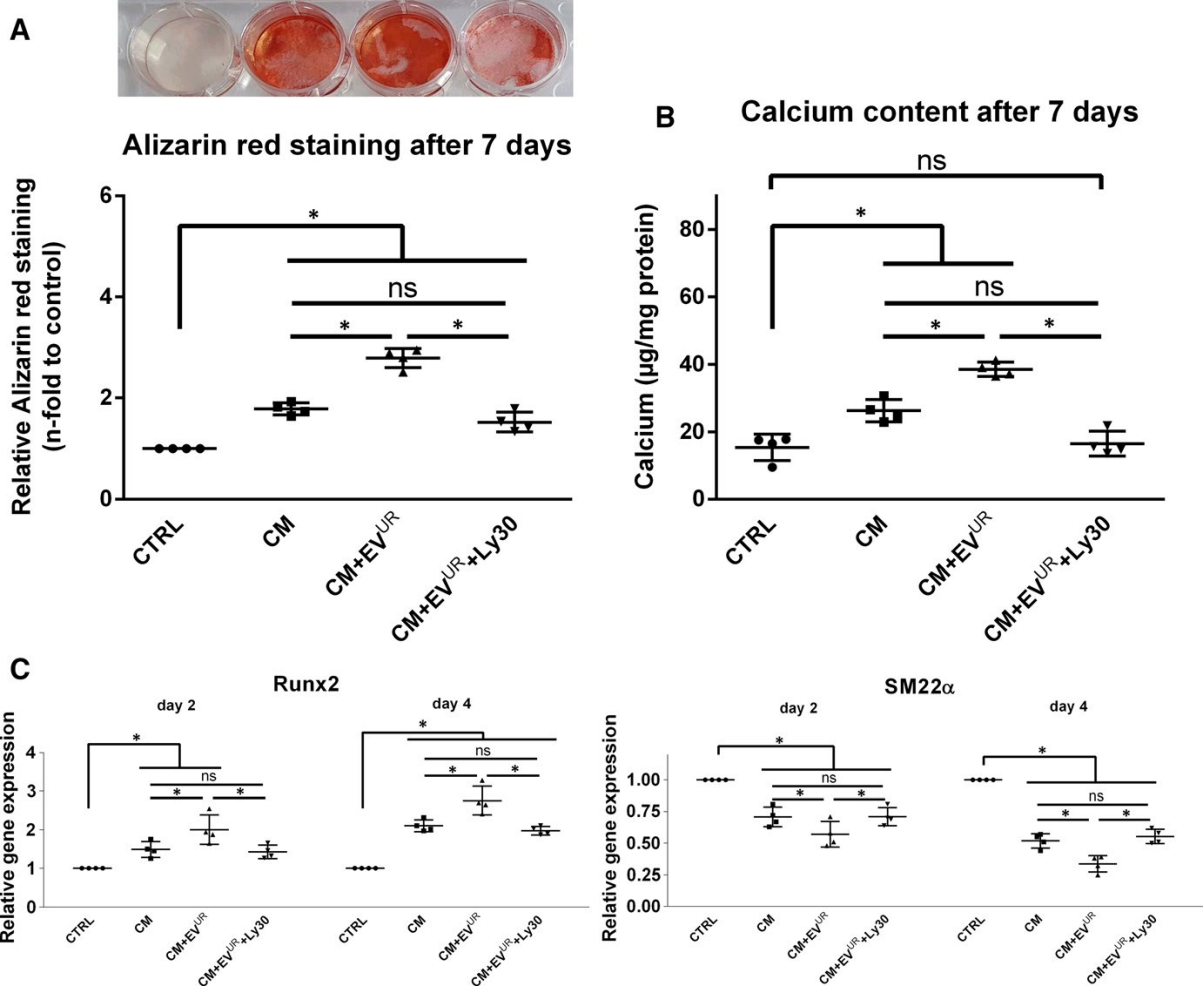
Inhibition of AKT signalling neutralizes pro‐calcifying effects of rat plasma‐derived EV^UR^ on osteogenic VSMC transdifferentiation. VSMC were treated like in the previous calcification experiments. This time with or without the presence of the PI3K/AKT/mTOR inhibitor Ly3023414 (Ly30). A, After 7 days, the cells were stained with Alizarin‐red. Shown are representative images of stained cells together with means ± SD from quantification of the red dye (n = 4). B, In addition, calcium contents were determined by the o‐cresolphtalein method (n = 4). C, Finally, effects of Ly30 on the transcription of runx2 and SM22a in VSMC were determined by qPCR at the indicated time points. Shown are means ± SD (n = 4). *P <.05; ^ns^not significant

Like for the in vitro‐derived EV^UR^, we also tested whether inhibition of AKT signalling can neutralize pro‐calcifying effects of the in vivo‐derived EV^UR^. Indeed, the presence of Ly30 reduced the degree of mineralization of the VSMC cultures to the level induced by treatment with CM alone (Figure 7A,B). On transcriptional level, Ly30 also neutralized the EV^UR^‐mediated up‐regulation of runx2 and the down‐regulation of SM22a in VSMC (Figure 7C).

## 4 DISCUSSION

Our results indicate that exosome‐like extracellular vesicles derived from uraemic toxin‐treated endothelial cells (EV^UR^) in vitro, as well as plasma‐derived EV^UR^ from uraemic rats, could facilitate the calcium‐ and phosphate‐induced osteogenic transdifferentiation/calcification of VSMC. Thus, endothelial cell‐derived extracellular vesicles may act as endogenous amplifiers of vascular calcification during CKD.

Besides a disturbed mineral homeostasis, CKD patients have elevated serum levels of uraemic toxins such as indoxyl sulphate (IS), which associates with a cardiovascular phenotype in children with CKD^23^ and promotes vascular calcification by activating the PI3K/Akt/NF‐κB pathway in VSMCs.^24^ Further, the diminished excretion of water‐soluble urea causes endothelial dysfunction and activation of proatherogenic pathways^18^ and fosters protein carbamylation, which aggravates the calcification of VSMC.^25^

Uraemic toxins along with other compounds such as TNF‐α, glucose (high concentrations), thrombin and angiotensin II were shown to induce EV formation by endothelial cells.^26^ Further, physical aspects such as shear stress can impact the release of EV.^27^

In the present study, we applied IS and urea in our in vitro model with EC to mimic a basic uraemic milieu. To obtain resulting EV from EC supernatants, we set up a combined isolation procedure, which reliably enabled us to isolate a presumptive exosome‐containing fraction. A characterization of the isolated EV revealed an exosome‐like size^7^ and an expression of the exosome‐specific marker proteins CD9, CD81 and Alix.^28^ Thus, EV^CTRL^ and EV^UR^ displayed exosome‐like characteristics. However, further investigations would be necessary to verify the endosomal system‐derived origin of both vesicle fractions. Instead of exosomes, we therefore used the more general terms EV^CTRL^ or EV^UR^ for both “exosome‐like” fractions in this study.

Key events during the development of arteriosclerosis are the osteochondrogenic transdifferentiation and the entailed calcification of VSMC. Amongst others, these processes involve VSMC apoptosis, vesicle release and a shift in the balance of inhibitors and promoters.^2^, ^8^, ^9^

The transdifferentiated osteochondrogenic VSMC phenotype is characterized by enhanced expression of specific transdifferentiation markers and inhibition of smooth muscle cell lineage markers. Indeed, EV^UR^ accelerated and increased CM‐induced gene expressions of osterix, osteocalcin and runx2, typical markers for an osteogenic transdifferentiation of VSMC^29^, ^30^ and further decreased the gene expression of SM22α, a marker of the differentiated contractile VSMC phenotype.^29^ In addition, VSMC cultures displayed higher degrees of calcifications and their supernatants contained elevated levels of ALP, a marker for advanced osteogenic transdifferentiation of VSMC.^31^ This clearly implicates that EV^UR^ promotes the CM‐induced transdifferentiation/calcification of VSMC.

Promoting and accelerating effects on VSMC calcification have also been described earlier for other substances. TNF‐α was shown to activate NF‐κB in VSMC, thereby accelerating VSMC calcification induced by elevated concentrations of phosphate.^32^ Also, an overexpression of Prelamin A, the unprocessed form of the nuclear lamina protein lamin A, promotes osteogenic differentiation of VSMC.^33^ In both mentioned studies, a calcification medium with elevated phosphate concentrations, like in our model, was used. Also, high‐dose insulin was shown to accelerate VSMC calcifications induced by β‐glycerolphosphate.^34^ However, in this study the process of VSMC calcification takes longer than in our calcification model so that the effects of insulin were first evident after ~3 weeks.

First insights into underlying mechanisms of EV^UR^ in VSMC were obtained by Western blot experiments. Since the effects of EV^UR^ on transcription/osteogenic conversion of VSMC were restricted to the presence of CM (elevated concentrations essentially of phosphate), we investigated potential effects of EV^UR^ on the protein expression of the sodium‐dependent phosphate co‐transporter PiT‐1. PiT‐1 is a predominant phosphate transporter in rats^35^ and has been shown to be necessary for phosphate‐induced activation of VSMC transdifferentiation and calcification.^22^ Indeed, the supplementation of EV^UR^ to CM enhanced the protein expression of PiT‐1 in VSMC after 2 and predominantly after 5 days of treatment compared to CM treatment alone. Consequent siRNA experiments revealed that a knock down of PiT‐1 effectively neutralized the pro‐calcifying effects of EV^UR^. This implicates PiT‐1 as one target of EV^UR^ in VSMC and therefore as one mode of action of how EV^UR^ foster the calcification of VSMC.

PiT‐1 seems also necessary for extracellular signal‐regulated kinase (ERK)1/2 phosphorylation in VSMC.^36^ This activation of ERK1/2^37^, ^38^ and also of AKT^39^, ^40^, ^41^ impact VSMC calcification, for example, via regulating the key osteogenic transcription factor runx2.^40^, ^42^ Consistent with these findings, we could show that EV^UR^ stimulated the phosphorylation of ERK1/2 and predominantly of AKT in VSMC. Activating effects on ERK1/2 by endothelial cell‐derived exosomes has also been described for cardiomyocytes.^43^ Another example for the extracellular vesicles‐induced activation of AKT and ERK in VSMC is foam cell‐derived exosomes during the development of atherosclerosis.^44^

A logical step was to apply respective inhibitors of relevant signalling pathways in VSMC calcification experiments to elucidate their impact on the effects of EV^UR^. Indeed, previous studies showed that the inhibition of AKT was effective to inhibit VSMC calcification.^39^ In the present study, also the EV^UR^‐induced calcification and the aggravating effects on osteogenic transcription of VSMC were neutralized by pharmacological inhibition of AKT. In conjunction with the neutralizing effects by ERK inhibition in our experiments, these data confirmed the activation of AKT and ERK as intracellular targets for EV^UR^ during VSMC calcification.

Hitherto, we looked at EV^CTRL^ and EV^UR^ as ‘normal’ drugs. However, EV also comprise a milieu‐dependent loading, for example, with calcification inhibitors and microRNAs (miR), thereby functioning as carriers or communicators between cells and in that way affecting cellular behaviour including calcification of VSMC.^45^ Such a communication between EC and VSMC via EV has been described earlier. For instance, shear‐stress‐stimulated EC release exosomes that could contain the specific miR‐143/145, which post‐transcriptionally control the expressions of genes that regulate the acquisition of a contractile VSMC phenotype.^13^, ^46^

Aiming to obtain first insights into differences in the cargo load between EV^UR^ and EV^CTRL^, we analysed the abundance of five preselected miR which are all associated with CKD and cardiovascular disease. Indeed, we found that levels of the above‐mentioned miR‐143 and ‐145 were reduced in EV^UR^ compared to EV^CTRL^. Decreased levels of these miR have been found in CKD patients compared to controls and were associated with an osteoblast‐like VSMC phenotype and enhanced vascular calcification.^47^

In contrast, levels of miR‐126, ‐221 and ‐222 were enhanced in EV^UR^. These miR are involved in the pathophysiology of EC^48^ and impact the progression of cardiovascular disease. Of note, up‐regulation of miR‐221/222 in glioma cells resulted in enhanced phosphorylation of AKT.^49^

By applying specific miR inhibitors and miR mimics, we could prove that differences in miR loading between EV^UR^ and EV^CTRL^ contribute to the pro‐calcifying effects of EV^UR^. Concomitant treatment of EV^UR^ with inhibitors of miR‐221 and miR‐222 effectively blocked the aggravating effects of EV^UR^ on CM‐induced calcifications. A similar result was obtained by concomitant application of mimics for miR‐143 and miR‐145.

Thus, differences in the miR loading between EV^UR^ and EV^CTRL^ contribute to the observed effects in VSMC and demand further studies in the future. However, differences in miR loading cannot explain the relatively fast effects (~15 min) of EV^UR^ on the phosphorylation of AKT in VSMC. There are different approaches to explain this issue.^50^ For cancer cells, it is known that secreted EV can contain PIK3CA, a PI3K catalytic subunit, thereby stimulating proliferation in target cells.^51^ Further, EV from retinal pigment epithelial cells were shown to contain active phosphorylated proteins that can mediate PI3K/Akt signalling pathway.^52^ In addition, EV from gastric cancer increase the phosphorylation of Akt in target cells which could be inhibited by the PI3K/Akt inhibitor LY294002.^53^ Thus, EV could mediate PI3K/Akt signalling through transfer of membrane binding ligands and through activated phosphorylated proteins.^50^ This issue also demands further research in subsequent studies.

The validity of our experimental approach is supported by a recent study from Alique et al^54^ which demonstrated that IS‐treated EC produce larger microvesicles with pro‐calcifying effects on VSMC. In addition, Lin et al^55^ demonstrated that also high glucose‐stimulated EC release exosomes with pro‐calcifying effects on VSMC. To our knowledge, we here for the first time demonstrate pro‐calcifying effects of uraemic toxin‐induced smaller exosome‐like EV from EC in VSMC.

First own ex vivo experiments complemented our in vitro data. Plasma‐derived EV from uraemic rats also augmented the CM‐induced transdifferentiation/calcification of VSMC in vitro. Further, AKT inhibition neutralized the EV^UR^‐mediated effects. These data provide a further rationale to pursue the concept of EV^UR^ as potential regulators of the complex pathophysiology of vascular calcifications in subsequent in vivo studies.

In summary, our data suggest that during CKD, the uraemic milieu in the serum favours the release of a pro‐calcifying EV subtype. These ‘uraemic’ EV in turn can promote the phenotypic switch and the calcification of VSMC. A schematic representation is shown in Figure S6.

### 4.1 Limitations of the study

We used an artificial model system to study effects of extracellular vesicles on VSMC calcification. The complex uraemic milieu during CKD was only very basically simulated by two uraemic toxins and static conditions (no shear stress to EC). Despite several washing steps of the EV fractions, we cannot exclude that they are ‘contaminated’ with certain cytokines or growth factors. Further, the VSMC in our in vitro model might display a kind of half‐activated/transdifferentiated phenotype because of the 2D environment.^56^ In addition, we here studied extracellular vesicles of human origin in a rat cell culture model of vascular calcification. However, human exosomes were also applied in rats in vivo earlier showing that an interspecies‐specific interaction occurs.^57^

## CONFLICT OF INTEREST

The authors confirm that there are no conflicts of interest.

## AUTHOR CONTRIBUTIONS

**Christian Freise:** Conceptualization (lead); Data curation (lead); Formal analysis (lead); Investigation (lead); Methodology (lead); Project administration (equal); Writing‐original draft (lead); Writing‐review & editing (lead). **Uwe Querfeld:** Conceptualization (equal); Writing‐review & editing (supporting). **Antje Ludwig:** Data curation (equal); Writing‐review & editing (supporting). **Bernd Hamm:** Project administration (equal); Writing‐review & editing (supporting). **Jörg Schnorr:** Funding acquisition (lead); Project administration (equal); Supervision (lead); Writing‐review & editing (supporting). **Matthias Taupitz:** Formal analysis (supporting); Funding acquisition (lead); Project administration (lead); Supervision (equal); Writing‐review & editing (supporting).

## Supporting information

Fig S1

Fig S2

Fig S3

Fig S4

Fig S5

Fig S6

Table S1

Table S2

Appendix S1

## Data Availability

The data that support the findings of this study are available from the corresponding author upon reasonable request.
