# Supplementary material for: Uraemic extracellular vesicles augment osteogenic transdifferentiation of vascular smooth muscle cells via enhanced AKT signalling and PiT‐1 expression
Source: J Cell Mol Med. 2021 May 7;25(12):5602–14. doi: 10.1111/jcmm.16572 (PMC8184672; doi:10.1111/jcmm.16572)
Supplement: Supplementary file 1 — Fig S1 [file JCMM-25-5602-s009.docx]

Supporting Figure S1:

**Supporting Figure S1. Flow chart of the procedure to isolate extracellular vesicles from cell culture supernatants.** All isolation steps were performed at 4°C.

Supporting Figure S2:

**Supporting Figure S2. Characterization of the isolated EV**. (**A**) The mean sizes of EV^UR^ and EV^CTRL^ were determined by dynamic light scattering (DLS) measurements. Shown is a representative result from a DLS measurement and the respective means±SD from four independent measurements. *p<0.05. (**B**) The exosome-specific markers CD9 (Tetraspanin 29) and CD81 (Tetraspanin 28) on the isolated EV were detected by FACS analysis. Besides EV^UR^ and EV^CTRL^ from the putative “exosome”-fraction, EV^UR^ and EV^CTRL^ from the putative “microvesicle”-fraction were analyzed. Shown are representative data from three independent experiments. (**C**) Complementing the FACS data, the protein expression of the exosome-marker Alix in the four groups was determined by western blot analysis. Shown are representative datasets from two independent experiments (MV = putative microvesicle fraction, Exo = putative exosome fraction). *p<0.05.

Supporting Figure S3:

**Supporting Figure S3.** **EV^UR^ induce morphological and transcriptional changes in VSMC only when applied in calcification medium.** (**A**) Rat VSMC were treated as indicated with CM supplemented with or without EV^CTRL^ or EV^UR^ for the indicated time durations before digital photos were taken. Shown are representative images from one out of four independent experiments. Black arrows indicate treatment dependent changes of cellular morphology. (**B**) Rat VSMC were treated as indicated with standard culture medium supplemented with or without EV^CTRL^ or EV^UR^ for the indicated time durations before digital photos were taken. Shown are representative images from one out of four independent experiments. (**C**) Gene expressions in VSMC were determined by qPCR after treatment of the cells with standard culture medium supplemented with or without EV^CTRL^ or EV^UR^. Shown are means ± SD (n=4). *Bar, 100 µm.*

Supporting Figure S4:

**Supporting Figure S4. Effects of EV^UR^ on apoptosis in VSMC**. Cell cycle-synchronized VSMC were treated for 4 days as indicated. Enzymatic activities of caspase-3/-7 were determined from cell lysates by fluorogenic caspase substrate conversion as described [1]. Treatment with 10 nM staurosporine served as positive control for the induction of apoptosis. Shown are means ± SD (n = 3). Statistics were calculated using one-way ANOVA followed by the Tukey post hoc test. *p<0.05.

Supporting Figure S5:

**Supporting Figure S5. Control of the transfection efficiency of EV^UR/CTRL^ with miR mimics and miR inhibitors.** 24 h after transfection, miR contents in the exosomes were analyzed by RT-PCR measurements. The dotted line represents the respective miR levels after usage of negative controls for a miR inhibitor or a miR mimic. Shown are means ± SD (n = 3). Statistics were calculated using one-way ANOVA followed by the Tukey post hoc test. *p<0.05 (compared to respective miR CTRL).

Supporting Figure S6:

**Supporting Figure S6. Deduced postulation for the effects of EV^UR^ on vascular calcification.** Under physiological conditions, endothelial cells secrete extracellular vesicles that help to maintain a contractile phenotype in VSMC. During uremia, enhanced serum levels of uremic toxins induce a pro-calcifying subset of “uremic” exosome-like extracellular vesicles in EC (EV^UR^). In conjunction with enhanced serum levels of calcium and phosphate, EV^UR^ accelerate the osteogenic transdifferentiation/calcification of VSMC, thereby promoting vascular calcification. If verified in vivo, EV^UR^ might supplement the complex mechanistic network of vascular calcification in CKD.

**References**

1. **Freise C, Querfeld U.** Inhibition of vascular calcification by block of intermediate conductance calcium-activated potassium channels with TRAM-34. *Pharmacological research*. 2014; 85: 6-14.
