# Supplementary material for: Uraemic extracellular vesicles augment osteogenic transdifferentiation of vascular smooth muscle cells via enhanced AKT signalling and PiT‐1 expression
Source: J Cell Mol Med. 2021 May 7;25(12):5602–14. doi: 10.1111/jcmm.16572 (PMC8184672; doi:10.1111/jcmm.16572)
Supplement: Supplementary file 2 — Fig S2 [file JCMM-25-5602-s006.docx]

Supporting Figure S2:

**Supporting Figure S2. Characterization of the isolated EV**. (**A**) The mean sizes of EV^UR^ and EV^CTRL^ were determined by dynamic light scattering (DLS) measurements. Shown is a representative result from a DLS measurement and the respective means±SD from four independent measurements. *p<0.05. (**B**) The exosome-specific markers CD9 (Tetraspanin 29) and CD81 (Tetraspanin 28) on the isolated EV were detected by FACS analysis. Besides EV^UR^ and EV^CTRL^ from the putative “exosome”-fraction, EV^UR^ and EV^CTRL^ from the putative “microvesicle”-fraction were analyzed. Shown are representative data from three independent experiments. (**C**) Complementing the FACS data, the protein expression of the exosome-marker Alix in the four groups was determined by western blot analysis. Shown are representative datasets from two independent experiments (MV = putative microvesicle fraction, Exo = putative exosome fraction). *p<0.05.
