# Supplementary material for: Uraemic extracellular vesicles augment osteogenic transdifferentiation of vascular smooth muscle cells via enhanced AKT signalling and PiT‐1 expression
Source: J Cell Mol Med. 2021 May 7;25(12):5602–14. doi: 10.1111/jcmm.16572 (PMC8184672; doi:10.1111/jcmm.16572)
Supplement: Supplementary file 3 — Fig S3 [file JCMM-25-5602-s004.docx]

Supporting Figure S3:

**Supporting Figure S3.** **EV^UR^ induce morphological and transcriptional changes in VSMC only when applied in calcification medium.** (**A**) Rat VSMC were treated as indicated with CM supplemented with or without EV^CTRL^ or EV^UR^ for the indicated time durations before digital photos were taken. Shown are representative images from one out of four independent experiments. Black arrows indicate treatment dependent changes of cellular morphology. (**B**) Rat VSMC were treated as indicated with standard culture medium supplemented with or without EV^CTRL^ or EV^UR^ for the indicated time durations before digital photos were taken. Shown are representative images from one out of four independent experiments. (**C**) Gene expressions in VSMC were determined by qPCR after treatment of the cells with standard culture medium supplemented with or without EV^CTRL^ or EV^UR^. Shown are means ± SD (n=4). *Bar, 100 µm.*
