# Supplementary material for: Uraemic extracellular vesicles augment osteogenic transdifferentiation of vascular smooth muscle cells via enhanced AKT signalling and PiT‐1 expression
Source: J Cell Mol Med. 2021 May 7;25(12):5602–14. doi: 10.1111/jcmm.16572 (PMC8184672; doi:10.1111/jcmm.16572)
Supplement: Supplementary file 4 — Fig S4 [file JCMM-25-5602-s008.docx]

Supporting Figure S4:

**Supporting Figure S4. Effects of EV^UR^ on apoptosis in VSMC**. Cell cycle-synchronized VSMC were treated for 4 days as indicated. Enzymatic activities of caspase-3/-7 were determined from cell lysates by fluorogenic caspase substrate conversion as described [1]. Treatment with 10 nM staurosporine served as positive control for the induction of apoptosis. Shown are means ± SD (n = 3). Statistics were calculated using one-way ANOVA followed by the Tukey post hoc test. *p<0.05.

**References**

1. **Freise C, Querfeld U.** Inhibition of vascular calcification by block of intermediate conductance calcium-activated potassium channels with TRAM-34. *Pharmacological research*. 2014; 85: 6-14.
