# Supplementary material for: Uraemic extracellular vesicles augment osteogenic transdifferentiation of vascular smooth muscle cells via enhanced AKT signalling and PiT‐1 expression
Source: J Cell Mol Med. 2021 May 7;25(12):5602–14. doi: 10.1111/jcmm.16572 (PMC8184672; doi:10.1111/jcmm.16572)
Supplement: Supplementary file 5 — Fig S5 [file JCMM-25-5602-s001.docx]

Supporting Figure S5:

**Supporting Figure S5. Control of the transfection efficiency of EV^UR/CTRL^ with miR mimics and miR inhibitors.** 24 h after transfection, miR contents in the exosomes were analyzed by RT-PCR measurements. The dotted line represents the respective miR levels after usage of negative controls for a miR inhibitor or a miR mimic. Shown are means ± SD (n = 3). Statistics were calculated using one-way ANOVA followed by the Tukey post hoc test. *p<0.05 (compared to respective miR CTRL).
