# Supplementary material for: Uraemic extracellular vesicles augment osteogenic transdifferentiation of vascular smooth muscle cells via enhanced AKT signalling and PiT‐1 expression
Source: J Cell Mol Med. 2021 May 7;25(12):5602–14. doi: 10.1111/jcmm.16572 (PMC8184672; doi:10.1111/jcmm.16572)
Supplement: Supplementary file 6 — Fig S6 [file JCMM-25-5602-s002.docx]

Supporting Figure S6:

**Supporting Figure S6. Deduced postulation for the effects of EV^UR^ on vascular calcification.** Under physiological conditions, endothelial cells secrete extracellular vesicles that help to maintain a contractile phenotype in VSMC. During uremia, enhanced serum levels of uremic toxins induce a pro-calcifying subset of “uremic” exosome-like extracellular vesicles in EC (EV^UR^). In conjunction with enhanced serum levels of calcium and phosphate, EV^UR^ accelerate the osteogenic transdifferentiation/calcification of VSMC, thereby promoting vascular calcification. If verified in vivo, EV^UR^ might supplement the complex mechanistic network of vascular calcification in CKD.
