# Supplementary material for: Uraemic extracellular vesicles augment osteogenic transdifferentiation of vascular smooth muscle cells via enhanced AKT signalling and PiT‐1 expression
Source: J Cell Mol Med. 2021 May 7;25(12):5602–14. doi: 10.1111/jcmm.16572 (PMC8184672; doi:10.1111/jcmm.16572)
Supplement: Supplementary file 7 — Table S1 [file JCMM-25-5602-s007.docx]

Supporting Table S1

**Supporting Table 1.** Serum chemistry in uremic and control rats after 20 weeks of treatment (means±SD).

|  | **adenine (n=3)** | **control (n=3)** |
| --- | --- | --- |
| creatinine (µmol/l) | 94.6 ± 23.4 | 21.4 ± 2.9 |
| urea (mmo/l) | 23.37 ± 3.39 | 7.57 ± 0.18 |
| phosphate (mmol/l) | 3.37 ± 1.65 | 1.76 ± 0.24 |
| calcium (mmol/l) | 2.35 ± 0.38 | 2.50 ± 0.10 |
