# Supplementary material for: Uraemic extracellular vesicles augment osteogenic transdifferentiation of vascular smooth muscle cells via enhanced AKT signalling and PiT‐1 expression
Source: J Cell Mol Med. 2021 May 7;25(12):5602–14. doi: 10.1111/jcmm.16572 (PMC8184672; doi:10.1111/jcmm.16572)
Supplement: Supplementary file 8 — Table S2 [file JCMM-25-5602-s003.docx]

Supporting Table S2

**Supporting Table S2.** Shown are human miR-inhibitors, miR-mimics and respective controls that were used to transfect EV^UR^/^CTRL^.

| miRBaseID | miRBase Accession # / Product number # | Product type | Company |
| --- | --- | --- | --- |
| hsa-miR-221-5p | MIMAT0004568 | *mir*VANA® miRNA inhibitor | Thermofisher scientific |
| hsa-miR-222-3p | MIMAT0000279 | *mir*VANA® miRNA inhibitor | Thermofisher scientific |
| hsa-miR-143-5p | MIMAT0004599 | *mir*VANA® miRNA mimic | Thermofisher scientific |
| hsa-miR-145-5p | MIMAT0000437 | *mir*VANA® miRNA mimic | Thermofisher scientific |
| hsa-miR-126-5p | MIMAT0000444 | *mir*VANA® miRNA inhibitor | Thermofisher scientific |
|  | 4464076 | *mir*VANA^TM^ miRNA Inhibitor, Negative Control | Invitrogen^TM^ |
|  | 4464058 | *mir*VANA^TM^ miRNA Mimic, Negative Control | Invitrogen^TM^ |
