# Supplementary material for: Uraemic extracellular vesicles augment osteogenic transdifferentiation of vascular smooth muscle cells via enhanced AKT signalling and PiT‐1 expression
Source: J Cell Mol Med. 2021 May 7;25(12):5602–14. doi: 10.1111/jcmm.16572 (PMC8184672; doi:10.1111/jcmm.16572)
Supplement: Supplementary file 9 — Appendix S1 [file JCMM-25-5602-s005.docx]

**Supporting content: materials & methods**

**Isolation of extracellular vesicles from cell culture supernatants and plasma from rats:**

After treatment with the uremic toxins, the cells in the flasks were washed two times with sterile-filtered PBS and cultured for additional 24h in standard culture medium supplemented with 5% Exosome-Depleted FBS (Gibco) instead of “normal” FBS. The culture medium was then removed and centrifuged at 700 x g for 5 min to remove cells followed by centrifugation at 2000 x g for 20 min to remove cellular debris. The remaining supernatants were then concentrated using Vivaspin 20 centrifugal filter devices with 3K NMWL (GE Healthcare) at 3220 x g followed by centrifugation for 30 min at 20;000 x g to obtain the microvesicle fraction. The supernatant was diluted 1:2 with the Total Exosome Isolation (from cell culture media) Reagent (Invitrogen) and incubated on a rotary shaker for 18h at 4°C. The exosome fraction was obtained by centrifugation of the solution for 60 min at 10,000 x g. The pellet was washed twice with ice cold 0.9% NaCl solution (B. Braun Melsungen AG, Melsungen, Germany), resuspended in 100 µl ice cold 0.9% NaCl solution and used immediately or stored for further analyses and experiments at -80°C.

EV from rat plasma were isolated with the Total Exosome Isolation (from plasma) Reagent (Invitrogen) without Proteinase K treatment. The pellets were washed, pooled and stored in NaCl solution as described above. The protein contents of the isolated EV fractions were determined by the Pierce™ BCA™ Protein-Assay (Thermo Fisher Scientific). Briefly, the EV samples (dissolved in NaCl) were diluted 1:2 or 1:4 with H_2_O (depending on the size of the isolated EV pellets and were directly subjected to the BCA assay according to the manufacturer’s instructions. The quantification of EV referring to their concentration as total protein yielded reproducible results.

**Characterization of extracellular vesicles:**

**Analysis of hydrodynamic diameters by dynamic light scattering**

The hydrodynamic diameters of the isolated vesicles from cell supernatants and rat plasma were determined by dynamic light scattering. 5 µg vesicles were diluted in 65 µl of a 0.9% NaCl solution in disposable micro cuvettes (Malvern Instruments Ltd., UK). DLS measurements were conducted at 23°C using a Zetasizer Nano ZS (Malvern Instruments Ltd., UK), operated at 633 nm and backscattering at 173°. Light scattering was recorded for 10 x 12s with 8 replicate measurements for each sample.

**Analysis of exosome-specific markers by fluorescence-activated cell sorting**

The expressions of the exosome-specific marker proteins CD9 (Tetraspanin 28) and CD81 (Tetraspanin 29) were analyzed by fluorescence-activated cell sorting analysis according to [1]. The extracellular vesicles were coupled to large streptavidin-coated beads, which allows the individual detection of the vesicles on the flow cytometer by biotin-coated antibodies. Briefly, 100 µl PureProteome™ Streptavidin Magnetic Beads (Millipore, Merck KGaA, Darmstadt, Germany) were incubated with 5.5 µg anti-CD81-biotin and 5.5 µg anti-CD9-biotin antibodies (Biolegend, San Diego, CA, USA). The conjugated beads were washed with T-TBS and resuspended in 500 µl T-TBS. 50 µl of the beads were then incubated with 12.5 µg of the resuspended vesicle fractions for ~18 h at 4°C. The EV-coated beads were washed and resuspended in 100 µl blocking buffer (5 mg/ml casein, 25 mM Tris and 150 mM NaCl at pH 7.4). 1.25 µg Fc block (BD Biosciences, San Jose, CA, USA) was added for 10 min prior to the addition of the fluorescence-labelled antibodys at 10 μg/ml for 15 min (FITC anti-human CD9 and APC anti-human CD81, Biolegend). After a further washing step, the EV-bead conjugates were resuspended in 550 µl blocking buffer and subjected to FACS analysis on a BD Accuri C6 device (BD Biosciences).

**Analysis of the exosome-specific marker Alix by western-blot**

Finally, the protein expressions of the exosome-specific marker protein Alix in the isolated EV -fractions was determined by western blot. Briefly, the exosome pellets were lysed and equal protein amounts of lysates were subjected to western-blot as described [2]. Specific primary antibodies against Alix were purchased from from Cell Signaling, Beverly, MA, USA. Protein bands were visualized using the WesternBreeze® Chromogenic Immunodetection System (Invitrogen) and band densities were quantified using Image J software (version 1.50i; National Institutes of Health, Bethesda, MA, USA).

**Analysis of miRNA levels in EV by qRT-PCR**

miRNA from EV pellets were isolated using the miRNeasy micro kit (Qiagen, Hilden, Germany) according to the manufacturer's protocol. Isolated miRNAs were transcribed to cDNA using the TaqMan® Advanced miRNA cDNA Synthesis-Kit (Applied Biosystems, Inc., USA). The subsequent qRT-PCR was performed on a StepOne™ Real-Time PCR System device (Thermo Fisher Scientific) using the TaqMan® Universal PCR Master Mix II and TaqMan® Advanced MicroRNA Assays (Applied Biosystems). The miRNAs of interest were (all from Applied Biosystems): Hsa-mir-221-5p (478778_mir), hsa-mir-222-3p (477982_mir), Hsa-mir-126-3p (477887_mir), Hsa-mir-143-3p (477912_mir) and Hsa-mir-145-3p (477915_mir). The expression of U6 was used to normalize the miRNA expressions: U6 snRNA (001973). Expressions in uremic samples relative to controls were determined by using the 2^–∆∆Ct^ method.

**Rat model of chronic kidney disease:**

# All animal protocols were approved by the regional animal study committee of Berlin (Germany). The animals were kept in facilities with stable environmental conditions and a 12-hour artificial light day and night cycle. Experimental uremia in 9–10 weeks old male Wistar rats (Charles River, France) was induced by adenine feeding (0.3%; Altromin, Lage, Germany) for 4 weeks followed by feeding a high-phosphate diet (1.4% vs. 0.5%) for 16 weeks. Blood samples were collected at the end of the experiments and plasma samples were stored at -20°C. Serum chemistry characteristics of the experimental groups are given in supporting Table_S1.

**Analysis of gene and protein expressions in VSMC:**

Gene expressions in VSMC were determined by quantitative real-time reverse transcription–polymerase chain reaction (RT–PCR). VSMC were treated in parallel setups to the calcification measurements. Measurements were performed as described [[21](#_ENREF_21)]. Relative RNA amounts were calculated using the 2^−ΔΔCt^ method and normalized to mean mRNA expression of the housekeeping gene GAPDH. Primer sequences (Rattus Norvegicus) were: Runx2 (NM_001278483.1), 5’-GATGCCTTAGTGCCCAAATGT-3’, 3’-GGCTGAAGGGTGAAGAAAGC-5’; Osterix (Sp7, NM_001037632), 5’-CCCAACTGTCAGGAGCTAGA-3’, 3’-CCTCTTGCCACAGAAAAGC-5’, SM22α (Transgelin, NM_031549.2), 5’- CACCTATCCTCAGCCTCAGC-3’, 3’-TCCAAAGGACATTGGCTTCC-3’; BMP-2 (NM_017178.2), 5’-ACAACATGGAGATTGCGCTAA-3’, 3’- GTCAAGCCAAACACAAACAGC-5’ and GAPDH (NM_017008.4), 5’- GACAACTTTGGCATCGTGGAG-3’, 3’- ATGCAGGGATGATGTTCTGG-5’ (BioTez Berlin-Buch GmbH, Berlin, Germany).

To investigate treatment-dependent effects on signaling pathways, VSMC were serum-starved for 1 h prior to the treatment for 15 min similar to the calcification experiments. After 15 min, the cells were lysed and equal protein amounts of lysates were subjected to western-blot as described [[22](#_ENREF_22)]. To determine effects on other proteins, the cells were treated similar to the calcification experiments as indicated in the figures. Specific primary antibodies used were against extracellular-signal-regulated kinase (ERK)1/2 (#9102S), phospho-ERK1/2 (#4370S), AKT (#4691S) and phospho-AKT (#2965S, Cell Signaling, Beverly, MA, USA) as well as against alpha-smooth muscle actin (α-SMA, #A2547; Merck, Darmstadt, Germany), the sodium-dependent phosphate transporter PiT-1 (#GTX64727, GeneTex, Alton Pkwy Irvine, CA, USA) and β-Actin (#A5316, Merck). Protein bands were visualized using the WesternBreeze® Chromogenic Immunodetection System (Invitrogen). Band densities were quantified using Image J software (version 1.50i; National Institutes of Health, Bethesda, MA, USA).

**References**

1. **Morales-Kastresana A, Jones JC.** Flow Cytometric Analysis of Extracellular Vesicles. *Methods in molecular biology (Clifton, NJ)*. 2017; 1545: 215-25.

2. **Freise C, Querfeld U.** The lignan (+)-episesamin interferes with TNF-alpha-induced activation of VSMC via diminished activation of NF-kB, ERK1/2 and AKT and decreased activity of gelatinases. *Acta physiologica (Oxford, England)*. 2015; 213: 642-52.
